# A canine leishmaniasis pilot survey in an emerging focus of visceral leishmaniasis: Posadas (Misiones, Argentina)

**DOI:** 10.1186/1471-2334-10-342

**Published:** 2010-12-01

**Authors:** Israel Cruz, Lucrecia Acosta, Mariana N Gutiérrez, Javier Nieto, Carmen Cañavate, Jorge Deschutter, Fernando J Bornay-Llinares

**Affiliations:** 1 WHO Collaborating Centre for Leishmaniasis, Servicio de Parasitología, Centro Nacional de Microbiología, Instituto de Salud Carlos III, Ctra. Majadahonda-Pozuelo, km 2, 28220 Majadahonda-Madrid, Spain; 2 Área de Parasitología, Universidad Miguel Hernández, Ctra. de Valencia km 8.7, 03550 Elche-Alicante, Spain; 3 Cátedra de Parasitología, Facultad de Ciencias Exactas, Químicas y Naturales, Universidad Nacional de Misiones, 3300 Posadas, Misiones, Argentina; 4 Ministerio de Salud Pública de Misiones, 3300 Posadas, Misiones, Argentina

## Abstract

**Background:**

An increasing number of reports are calling our attention to the worldwide spread of leishmaniasis. The urbanization of zoonotic visceral leishmaniasis (VL) has been observed in different South American countries, due to changes in demographic and ecological factors. In May 2006, VL was detected for the first time in the city of Posadas (Misiones, Argentina). This event encouraged us to conduct a clinical and parasitological pilot survey on domestic dogs from Posadas to identify their potential role as reservoirs for the disease.

**Methods:**

One hundred and ten dogs from the city of Posadas were included in the study. They were selected based on convenience and availability. All dogs underwent clinical examination. Symptomatology related to canine leishmaniasis was recorded, and peripheral blood and lymph node aspirates were collected. Anti-*Leishmania *antibodies were detected using rK39-immunocromatographic tests and IFAT. Parasite detection was based on peripheral blood and lymph node aspirate PCR targeting the *SSUrRNA *gene. Molecular typing was addressed by DNA sequence analysis of the PCR products obtained by *SSUrRNA *and ITS-1 PCR.

**Results:**

According to clinical examination, 69.1% (76/110) of the dogs presented symptoms compatible with canine leishmaniasis. Serological analyses were positive for 43.6% (48/110) of the dogs and parasite DNA was detected in 47.3% (52/110). A total of 63 dogs (57.3%) were positive by serology and/or PCR. Molecular typing identified *Leishmania infantum *(syn. *Leishmania chagasi*) as the causative agent.

**Conclusions:**

This work confirms recent findings which revealed the presence of *Lutzomyia longipalpis*, the vector of *L. infantum *in this area of South America. This new VL focus could be well established, and further work is needed to ascertain its magnitude and to prevent further human VL cases.

## Background

In South America *Leishmania infantum *(syn. *Leishmania chagasi*) is the causative agent of visceral leishmaniasis (VL), a systemic infection which is fatal if not treated. The primary vectors are *Lutzomyia longipalpis *female sand flies, and infected domestic dogs are the main reservoirs [1]. However, Dantas-Torres recently highlighted the need to use proper diagnostic tools to identify the species of *Leishmania *involved in each case of canine leishmaniasis irrespective of the clinical form [2]; this is an important issue to take into account when a new focus is being described.

The worldwide incidence of VL is estimated to be 500000 cases/year, with more than 50000 related deaths. In several areas of the world, there is a clear and disturbing increase in the number of VL cases. For example, in Northeastern Brazil the incidence raised from 1840 cases in year 1998 to 6000 in 2002 [3].

At present, a growing number of reports are calling our attention to a worldwide spread of leishmaniasis, including the urbanization of VL in different South American countries due to changes in demographic and ecological factors [4-8].

An example of the latter is the recent report by Salomón, *et al. *[9] on the first urban VL focus in Posadas (Misiones, Argentina), which appeared in 2006 involving humans and dogs. That work indicated both: i) the presence of *Lu. longipalpis *in Misiones, where it was reported previously to be anecdotal [10] and ii) the presence of *Leishmania sp. *infection in 13 out of 27 dogs studied [9]. At present, 58 human VL cases have been reported in Posadas (2 in year 2006, 14 in 2007, 17 in 2008, 24 in 2009 and one up to April 30^th ^2010), with 6 related deaths [[11], Misiones Ministry of Health personal communication].

Encouraged by the emergence of the first human VL cases, we conducted a canine serological and parasitological pilot survey to: i) identify *Leishmania *infection in dogs, and thus their possible role as reservoirs, and ii) identify de *Leishmania *species circulating in this emerging VL focus.

## Methods

### Study location

The serological and parasitological pilot survey was conducted from 1 July to 15 August 2006 in the city of Posadas (27°23'S, 55°54'W), located in the southwest of Misiones province, Northeast Argentina.

Misiones is bordered by Brazil to the north and by Paraguay to the east. The city of Posadas has a global surface of 324 Km^2^, and is characterized by a subtropical humid climate; annual rain is 1700 mm and average temperature 21.5°C. Posadas, which accounts for 29% of the total population of the province, had an estimated population of 297499 inhabitants in 2008; 98.8% from urban areas and 32.6% below the poverty line [12].

### Study animals and samples

Our study was based on a convenience sample of 110 dogs originating from two sources: i) 83 dogs from two different canine shelters, located in the outskirts of Posadas, which admit dogs from the city: 59 from the non-profit civil association 'El Refugio' (Itaembé Miní area) and 24 from the Municipal Animal Health Institute-IMUSA (El Zaimán area), and ii) 27 dogs with owners that attended a local veterinary clinic for routinely care.

All dogs underwent clinical examination by a local veterinarian, searching for symptoms related to canine leishmaniasis (CanL). The presence of one or more of the following was considered for the clinical diagnosis of CanL: lymphadenopathy, onychogryphosis, skin lesions, weight loss, conjunctivitis and alopecia.

After examination one ml peripheral blood (PB) was obtained from 110 dogs and collected in EDTA-containing tubes; lymph node aspirates (LN) were obtained from 94 dogs and further diluted in 500 μl ethanol 70%. Once obtained, samples were stored at 4°C until shipment to the WHO Collaborating Centre for Leishmaniasis (Madrid, Spain) for serological and molecular diagnosis.

Informed consent was obtained from each dog owner and from the canine shelter responsible before clinical examination and sampling of the dogs. Research procedures were approved by the corresponding research review boards of Universidad Miguel Hernández and Misiones Ministry of Public Health.

### Serological diagnosis

Anti-*Leishmania *antibodies detection was performed by two different approaches: a) rK39-immunochromatographic test (rK39-ICT; Kalazar Detect™ Rapid test, InBIOS International, Seattle, WA), using 25 μl of peripheral blood and following the manufacturer's instructions; and b) Immunofluorescence antibody test (IFAT) following a standard method, using 10 μl of 2 × 10^7 ^*L. infantum *promastigotes/ml in 1× PBS per well as antigen (reference strain MHOM/FR/78/LEM-75) and 1 μl plasma for the analysis. The IFAT threshold titre for positivity was 1/160 [13]. Considering the present work a pilot study, replicate testing of the samples was not performed.

### Molecular diagnosis

Parasite detection was done by means of nested-PCR targeting the *Leishmania SSUrRNA *gene (LnPCR) as described elsewhere, this protocol is *Leishmania *genus specific and uses the primer pair R221 (5'-GGT TCC TTT CCT GAT TTA CG-3') and R332 (5'-GGC CGG TAA AGG CCG AAT AG-3') in the first reaction, and the primer pair R223 (5'-TCC CAT CGC AAC CTC GGT T-3') and R333 (5'-AAA GCG GGC GCG GTG CTG-3') in the nested reaction [14]. Starting material for DNA extraction was: i) 100 μL PB and ii) the pellet obtained after centrifugation (6000 rpm in a benchtop microcentrifuge for 10 min) and PBS 1× washing of the LN dilution obtained from each dog. DNA was extracted by conventional phenol-chloroform extraction and ethanol precipitation and further eluted in 100 μl sterile distilled water.

### Molecular typing of the parasites

*Leishmania *molecular typing was carried out by sequence analysis of both the LnPCR products obtained from PB and LN samples, and the ITS-1 PCR products obtained from PB samples of LnPCR positive dogs. ITS-1 PCR was performed as described elsewhere with the primer pair LITSR (5'-CTG GAT CAT TTT CCG ATG-3') and L5.8S (5'-TGA TAC CAC TTA TCG CAC TT-3') and used only for typing purpose [15]. Direct sequencing of the PCR products was performed with forward and reverse primers; using the Big-Dye Terminator Cycle Sequencing Ready Reaction Kit V3.1 and the automated ABI PRISM 377 DNA sequencer (Applied Biosystems, Foster City, CA). Sequences obtained were analyzed and edited using Lasergene^® ^sequence analysis software (DNASTAR, Madison, WI).

### Statistical analyses

Data recorded during the clinical examination and results from serological analyses were introduced in an Excel^® ^data sheet (Microsoft Office 2003). The association between the different variables was analyzed with the SPSS statistical software version 16.0, using the chi-square test with Yate's correction. A p-value of <0.05 was considered as statistically significant.

## Results

### Clinical examination

Dogs were grouped according to their age in four different groups. Group 1 (≤1 year) consisted in 23/110 dogs (20.9%), group 2 (2-5 years) in 55/110 (50.0%), group 3 (6-10 years) in 18/110 (16.4%), and group 4 (>10 years) in 14/110 (12.7%). Male/female ratio for all groups together was 1.5/1 (66 male/44 female). Eighty-three out of the 110 dogs (75.4%) were mongrel and 27/110 (24.6%) were from different breeds.

After clinical examination, 34 out of 110 dogs (30.9%) were classified as asymptomatic, while 76/110 (69.1%) presented one or more clinical signs related to canine leishmaniasis. The frequency of the different symptoms in the 76 symptomatic dogs is presented in table 1. Nineteen out of the 76 symptomatic (25%) dogs were classified as oligosymptomatic (1 or 2 symptoms), while 57 (75%) were classified as polysymptomatic (more than 3 symptoms).

**Table 1:** Frequency of signs/symptoms related to canine leishmaniasis in 76 symptomatic dogs.

Symptoms	Frequency (%)
Skin lesions	73/76 (96.0%)
Lymphadenopathy	68/76 (89.5%)
Onychogryphosis	47/76 (61.8%)
Weight loss	38/76 (50.0%)
Conjunctivitis	32/76 (42.1%)

### Serological and molecular diagnosis

All dogs were analyzed by rK39-ICT and/or IFAT. rK39-ICT was positive in 42/109 (38.5%) of the dogs, and IFAT in 40/102 (39.2%). The combination of both serological methods indicated that 48/110 (43.6%) of them were seropositive. For 101 dogs tested by both serological methods, the concordance between rK39-ICT and IFAT results was 90.1%. Dogs were considered as seropositive when rK39-ICT and/or IFAT yielded a positive result.

All dogs were analysed by PB-LnPCR and/or LN-LnPCR. *Leishmania *DNA was detected in PB of 23/109 (21.1%) and in LN of 47/94 (50.0%) of the dogs studied. The combination of both PCR approaches detected the parasite DNA in 52/110 (47.3%) dogs. For 93 dogs on which PCR was performed on both biological samples the concordance of PCR results was 63.4%. Dogs were considered as parasite positive when leishmanial DNA was detected by PB-LnPCR and/or LN-LnPCR.

Dogs were considered as infected when they were seropositive and/or parasite positive. Table 2 provides a detailed description of serology and PCR results with regards to the origin, sex, breed, age group and clinical status of 110 dogs on which both serology and PCR data were available.

**Table 2:** Detailed description of serology and PCR results with regards to different parameters in 110 dogs.

		Sero-pos*	Parasite-pos**	Infected***
**Origin**	Owner (N = 27)	14 (51.8%)	14 (51.8%)	18 (66.7%)
	Canine shelter (N = 83)	34 (40.9%)	38 (45.8%)	45 (54.2%)

**Sex**	Male (N = 66)	30 (45.4%)	32 (48.5%)	40 (60.6%)
	Female (N = 44)	18 (40.9%)	20 (45.4%)	23 (52.3%)

**Breed**	Defined breed (N = 27)	16 (59.2%)	11 (40.7%)	16 (59.2%)
	Mongrel (N = 83)	32 (38.5%)	41 (49.4%)	47 (56.6%)

**Age Group**	Group 1 (N = 23)	12 (52.2%)	12 (52.2%)	15 (65.2%)
	Group 2 (N = 55)	24 (43.6%)	29 (52.7%)	34 (61.8%)
	Group 3 (N = 18)	5 (27.8%)	7 (38.9%)	7 (38.9%)
	Group 4 (N = 14)	7 (50.0%)	4 (28.6%)	7 (50.0%)

**Clinical status**	Symptomatic (N = 76)	39 (51.3%)	42 (55.3%)	50 (65.8%)
	Asymptomatic (N = 34)	9 (26.5%)	10 (29.4%)	13 (38.2%)

**Total**	(N = 110)	48 (43.6%)	52 (47.3%)	63 (57.3%)

*Sero-pos: seropositive by rK39-ICT and/or IFAT. **Parasite-pos: LnPCR positive on PB and/or LN samples. ***Infected: Sero-pos and/or Parasite-pos.

**Figure 1:**
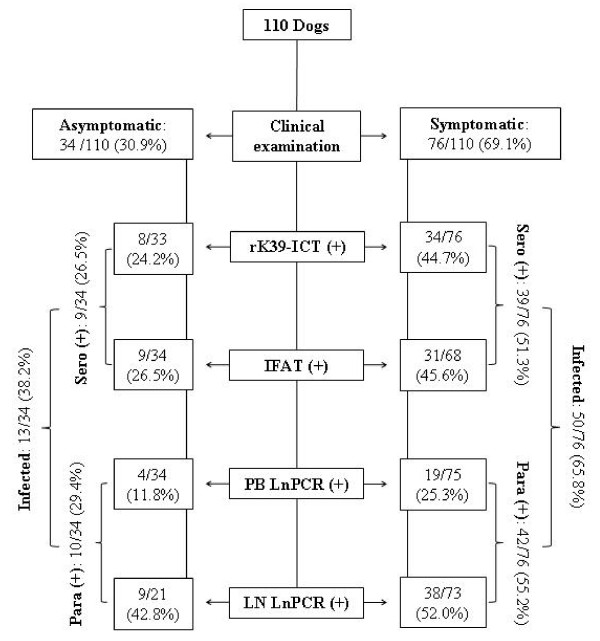
**Flowchart including data on clinical examination, the number of samples processed by each diagnostic test and those testing positive**. Flowchart including data on clinical examination, and number of samples processed. Sero (+): seropositive by rK39-ICT and/or IFAT. Para (+):LnPCR positive on PB and/or LN samples. Infected: Sero (+) and/or Para (+).

Figure 1 shows a flowchart including data on clinical examination, the number of samples processed by each diagnostic test and those testing positive. The number of dogs positive with one, two, three or four diagnostic approaches according to their clinical status is shown in Table 3.

**Table 3:** Number of dogs analyzed by all 4 diagnostic methods testing positive with one, two, three or four diagnostic approaches (rK39-ICT, IFAT, PB-LnPCR and LN-LnPCR) according to their clinical status.

Number of positive tests	Total N = 85 (%)	Asymptomatic N = 20 (%)	Symptomatic N = 65 (%)
**0**	32/85 (37.6)	11/20 (55.0)	21/65 (32.3)

**1**	14/85 (16.4)	3/20 (15.0)	11/65 (16.9)

**2**	10/85 (11.7)	1/20 (5.0)	9/65 (13.8)

**3**	15/85 (17.6)	2/20 (10.0)	13/65 (20.0)

**4**	14/85 (16.4)	3/20 (15.0)	11/65 (16.9)

### Molecular typing of the parasites

DNA sequences from LnPCR products were obtained for 23 PB and 45 LN samples from 53 different dogs. And DNA sequences from ITS-1 PCR products were obtained for PB samples from 17 different dogs. Once edited the sequences obtained were used for BLAST search against GenBank™ database [16]. The sequences obtained from the LnPCR products returned 100% identity with *SSUrRNA *gene sequences of *Leishmania donovani *complex species (*L. infantum*, *L. donovani*), while the sequences obtained from the ITS-1 PCR products returned 100% identity with ITS-1 sequences of *L. infantum*.

### Statistical analyses

No statistical association was observed between infection and age group (p = 0.279), sex (p = 0.387) or origin (p = 0.256). However there was a significant association between symptomatology and infection (p = 0.007). Furthermore, a significant association between number of symptoms and infection (p = 0.019) was observed. Particularly, the presence of the following symptoms presented significant association with infection: lymphadenopathy, onychogryphosys and conjunctivitis (p < 0.05); while skin lesions and weight loss were not associated (p > 0.05).

## Discussion

*Leishmania *infection was confirmed either by molecular and/or serological methods in 63/110 (57.3%) dogs. Current entomological data reporting the presence of *Lu. longipalpis *in Posadas [9], together with the presence of *L. infantum *infection in urban dogs indicates that the transmission cycle of zoonotic VL (ZVL) could be well established, and that further cases of human VL are likely to appear. It has been shown in Brazil that human epidemics of VL are usually preceded, or concomitant to high infection rates in the canine population [17-19]. The emergence of 58 human VL cases in Posadas since 2006 supports this possibility. The limitations of this pilot study, based on a convenience sampling, do not allow drawing solid conclusions. In addition most of the dogs (83/110) were from a canine shelter, thus the data do not have the power of a population-based random sampling study to provide a view of the real prevalence of *Leishmania *infection in the canine population. However the data present a consistent picture of this emerging focus of ZVL.

Infected symptomatic dogs are considered to be the main reservoirs of ZVL leishmaniasis, in the present study these represent 45.4% (50/110) of the dogs studied. However, infected asymptomatic dogs are also said to play a role in transmission [20-22], in this study these represent 11.8% (13/110) of the total. In spite of the statistical association between infection and symptomatology, the present study also highlights the low specificity of the clinical diagnosis of CanL; in fact 34.2% (26/76) of the symptomatic dogs (one or more of the before mentioned symptoms) presented negative results for both serology and PCR. Thus, as suggested by recent reviews, reliable laboratory-based diagnostic tests should be carried out either in clinical practice or in epidemiological studies [23,24].

In areas where ZVL is endemic the prevalence of infected dogs tends to be high, with a greater proportion of asymptomatic ones [25]. In the present study, and unlike what happens in traditional endemic areas for ZVL, most of the infected dogs were symptomatic. This could be due to a recent introduction of *L. infantum *in the area where, as a naïve population, most of the dogs infected would develop the disease. It is also reported in ZVL endemic areas an increasing prevalence of seropositive dogs with age, and a final decrease in those aged ≥7 years [26-28]. In dogs from the present study we observed no increase in seropositivity nor in parasite DNA detection related with the age of the dogs (p > 0.05). This finding could also be consistent with the hypothesis of a recent introduction of *L. infantum*. The results of the diagnostic tests employed also support the above mentioned. In the recent review by Baneth, *et al. *[29] it is stated that in endemic foci the number of PCR positive dogs will exceed the number of seropositive; however in the present study there were no great differences between the rates of infection detected by serology and PCR.

Concerning the serological test employed in this study (rK39-ICT and IFAT) they have presented a concordance of 90.1%. IFAT has long been considered as the *gold standard *in canine leishmaniasis serodiagnosis [1,30], and different rK39 rapid tests have also shown a good performance in field studies [31]. According to different authors their sensitivity ranges from 85.5 to 90% for IFAT and 72 to 96.7% for rK39-ICT, while their specificity ranges from 94.7 to 100% for IFAT and 61.75-100% for rK39-ICT [24,31-33]. However, Mettler *et al *[33] reported a lower sensitivity for these methods in asymptomatic infected dogs, 29.4% for IFAT and 52.9% for rK39-ICT. In our study no big differences were observed in the performance of these tests between asymptomatic and symptomatic dogs; In PCR-positive asymptomatic dogs rK39-ICT and IFAT were positive for 55.5% and 60% of the dogs respectively, while in PCR-positive symptomatic dogs both methods were positive for 66.6% of the dogs. Cross reactions with other infectious agents such as *Babesia canis*, *Ehrlichia canis*, *Neospora caninum*, *Hepatozoon canis *and *Trypanosoma cruzi *have also been described [31,33,34]. Some false-positive results of rK39-ICT have been also attributed to unknown blood factors present in dog blood [32]. Although we cannot categorically rule out cross-reactions with *T. cruzi *or *L. braziliensis *in our study, this is very unlikely as the presence of autochthonous infections by these parasites has not been reported in the city of Posadas, where this study took place.

With regards to the molecular typing of the parasites this is, to our knowledge and taking into account the review by Salomón *et al. *[9], the first report on molecular identification of autochthonous *L. infantum *infection in Argentina.

The recently established focus of human and canine VL in Asunción, Paraguay [35,36], could have been, because of its proximity, the source of the introduction of VL in Northern Argentina [37]. To ascertain the origin of the parasites in this new VL focus a wide population genetics based study involving *L. infantum *strains from different South American endemic areas could be very helpful. For this purpose multi locus microsatellite typing seems to be the most appropriate tool [38]. And to ascertain since when it is established, powerful epidemiological studies in human and canine population must be done. These studies should address: i) retrospective analyses of human and canine samples from patients/dogs attending the different health/veterinary centres in the area with symptoms compatible with human VL/CanL; ii) leishmanin skin test (in humans) and serological surveys (in both humans and dogs) to estimate the prevalence of parasite contact in different age groups.

## Conclusions

This pilot study confirms the presence of CanL due to *L. infantum *in Posadas (Misiones, Argentina), an area where the disease has been recently reported. Though the present work has the limitations of not being a population-based random sampling study, and does not provide an accurate view of the real prevalence of *Leishmania *infection in the canine population of Posadas, it indicates that this new VL focus could be well established, and further work is needed to ascertain its magnitude and to prevent further human VL cases.

## Competing interests

The authors declare that they have no competing interests.

## Authors' contributions

IC, LA, FJBL and JD conceptualized and designed the study. IC and LA drafted the manuscript; CC critically reviewed it and contributed to its design. FJBL coordinated the study. LA, MNG and JD contacted the dog owners and canine shelter that participated in the study. LA and MNG carried out clinical examination and obtained biological samples from the dogs. JN and MNG designed the protocol for clinical scoring of the dogs. IC, LA, JN and CC performed serological and molecular diagnosis. IC performed molecular typing of the parasites. All authors participated in the analysis and interpretation of data, revised the different draft versions, and read and approved the final manuscript.

## Pre-publication history

The pre-publication history for this paper can be accessed here:

http://www.biomedcentral.com/1471-2334/10/342/prepub
